# Clinical Evaluation of Paraspinal Mini-Tubular Lumbar Decompression and Minimally Invasive Transforaminal Lumbar Interbody Fusion for Lumbar Spondylolisthesis Grade I with Lumbar Spinal Stenosis: A Cohort Study

**DOI:** 10.3389/fsurg.2022.906289

**Published:** 2022-05-10

**Authors:** Zeyan Liang, Xiongjie Xu, Jian Rao, Yan Chen, Rui Wang, Chunmei Chen

**Affiliations:** Department of Neurosurgery, Fujian Medical University Union Hospital, Fuzhou, China

**Keywords:** degenerative lumbar spondylolisthesis, paraspinal mini-tubular lumbar decompression, minimally invasive transforaminal lumbar interbody fusion, lumbar spinal stenosis, minimally invasive spine surgery

## Abstract

**Objective:**

To investigate the clinical outcome data and difference in efficacy between paraspinal mini-tubular lumbar decompression (PMTD) and minimally invasive transforaminal lumbar interbody fusion (MIS TLIF) in the treatment of degenerative lumbar spondylolisthesis grade I with lumbar spinal stenosis (DLS-I-LSS).

**Methods:**

Patients with DLS-I-LSS, who underwent PMTD or MIS TLIF from September 2017 to March 2020, were included retrospectively. The follow-up period was 24 months after surgery. Outcome measurements included the Oswestry disability index (ODI) score, visual analog scale (VAS) low back pain score, VAS leg pain score, surgical data, and adverse events.

**Results:**

A total of 104 patients with DLS-I-LSS were included in this study. The average improvement in ODI at 12 months (2.0%, 95% CI, −5.7% to 1.8%; *p* = 0.30) and 24 months (1.7%, 95% CI, −2.7% to 6.1%; *p* = 0.45) after surgery between the two groups were not statistically significant. The improvement in VAS low back pain score after 24 months and improvement in VAS leg pain score were not significantly different between the two groups. Compared with the PMTD group, the MIS TLIF group had more estimated blood loss and longer hospital stays. The cumulative reoperation rates were 5.66% and 1.96% in the MIS TLIF and PMTD groups, respectively (*p* = 0.68). The results of multivariate analysis showed that BMI, diabetes, and baseline ODI score were the main factors influencing the improvement in ODI in patients with DLS-I-LSS after minimally invasive surgery, accounting for 50.5% of the total variance.

**Conclusions:**

The clinical effectiveness of PMTD was non-inferior to that of MIS TLIF for DLS-I-LSS; however, there was a reduced duration of hospital stay, operation time, blood loss, and hospitalization costs in the PMTD group. BMI, presence or absence of diabetes and baseline ODI score were influencing factors for the improvement of ODI (Trial Registration: ChiCTR2000040025).

## Introduction

Degenerative lumbar spondylolisthesis (DLS) is a spine disease that results in lower back pain (1–3). Patients with symptomatic lumbar spondylolisthesis may begin with conservative treatment strategies and physical rehabilitation training, including constrained motion, epidural steroid injection, and electrophotoluminescence (4–7). Surgical management is recommended in patients who fail conservative treatment strategies (6, 8). Decompression or decompression with fusion are the two main surgical options for DLS (9, 10). Recent evidence suggests that surgical treatment for DLS is superior to nonsurgical treatment (11, 12).

The main goal of surgery is to decompress the central canal, lateral recess, and nerve foramen for lumbar spinal stenosis associated with DLS (4). At present, whether additional internal fixation fusion should be performed after decompression in patients with degenerative lumbar spondylolisthesis grade I with lumbar spinal stenosis (DLS-I-LSS) remains controversial. In 2016, two prospective randomized controlled clinical studies of DLS-I-LSS were published in the New England Journal of Medicine. Forsh et al. (13) found that the effect of decompression with fusion was not better than that of decompression alone. However, Ghogawala et al. (14) indicated that decompression with fusion was superior to decompression alone. After combining the results of the two studies, decompression alone in the treatment of DLS-I-LSS may be as effective as decompression with fusion. At present, the most common surgical approach for lumbar spinal decompression is posterior midline laminectomy assisted microscopically (15).

In 1997, Foley and Smith independently reported the first microendoscopic discectomy (16). In 2002, Greiner-Perth et al. (17) reported the use of a microscope in combination with a channel system to address two-dimensional visual fields for the treatment of lumbar disc herniation (LDH). In China, Chunmei et al. (18, 19) were the first to combine a microscope with a microtube working system using a paraspinal approach to achieve bilateral decompression via a unilateral approach. Thus, the efficacy and safety of paraspinal mini-tubular lumbar decompression (PMTD) for the treatment of lumbar spinal stenosis were verified. Compared with the traditional posterior midline approach for spinal decompression, the surgical approach of PMTD is a paravertebral interlaminar approach, which preserves the integrity of the spinal muscles and ligaments based on expansion and blunt muscle separation. Therefore, PMTD has the potential to be as effective as decompression with fusion for patients with DLS-I-LSS (20).

Transforaminal lumbar interbody fusion (TLIF) is the most commonly used surgical procedure for nerve decompression and bone stabilization (21–24). Minimally invasive transforaminal lumbar interbody fusion (MIS TLIF) may result in spinal cord decompression and intervertebral fusion based on a mini-tubular approach and percutaneous pedicle screw placement (25–28).

At present, PMTD and MIS-TLF have been widely used for the treatment of DLS-I-LSS (29, 30). However, differences in efficacy and safety between the two surgical procedures have not been reported. This ambidirectional cohort study aimed to investigate the difference between PMTD and MIS TLIF in the treatment of DLS-I-LSS.

## Materials and Methods

### Patient Population

This ambidirectional cohort study was conducted at Fujian Medical University Union Hospital. After obtaining approval from the ethics board at Fujian Medical University Union Hospital (Ethics Approval Number, 2020KY0134) and registering the study at the Chinese Clinical Trial Registry (Clinical Study Registration Number, http://www.chictr.org.cn/, ChiCTR2000040025), we reviewed all patients with DLS-I-LSS who received PMTD or MIS TLIF performed by a spine neurosurgeon from September 2017 to March 2020. The Strengthening the Reporting of Observational Studies in Epidemiology (STROBE) recommendations were strictly followed in the reporting of this comparative study (31). The diagnostic criteria were as follows: (1) typical clinical manifestations: low back pain, leg pain, and intermittent claudication; (2) lumbar radiographs indicated grade I lumbar spondylolisthesis (according to the Meyerding classification (32)); (3) lumbar spondylolisthesis and lumbar spinal stenosis were confirmed by MRI and CT in all patients, and the stenosis location was consistent with the corresponding neurological symptoms. The inclusion and exclusion criteria are listed in Table 1.

**Table 1:** Inclusive and exclusive criteria.

**Inclusive criteria**
Age between 30 and 70 years
Typical clinical manifestations (eg. low back pain, leg pain, and intermittent claudication) with failed conservative treatment at least 3 months
Grade I lumbar spondylolisthesis (according to the Meyerding classification)
Symptoms are confirmed by CT and MRI, and matches the affected segment
Without lumbar instability
Received PMTD or MIS-TLIF
**Exclusive criteria**
Previous surgery on the same or adjacent segment
Multiple spondylolisthesis
Other serious physical, psychological or mental diseases
Currently participating in other clinical trials
Similar symptoms that caused by severe somatic or psychiatric illness
With a history of spinal cord injury/trauma
Cauda equina syndrome

*Preoperative hyperextension and flexion radiographs showed an angle difference of less than 10° between the upper and lower endplates of the affected segments or a transitional distance of less than 3 mm between the vertebral bodies.*

Patients were allocated to the PMTD or MIS TLIF group according to the actual conditions of the surgical procedure.

### Intervention: PMTD

**Figure 1:**
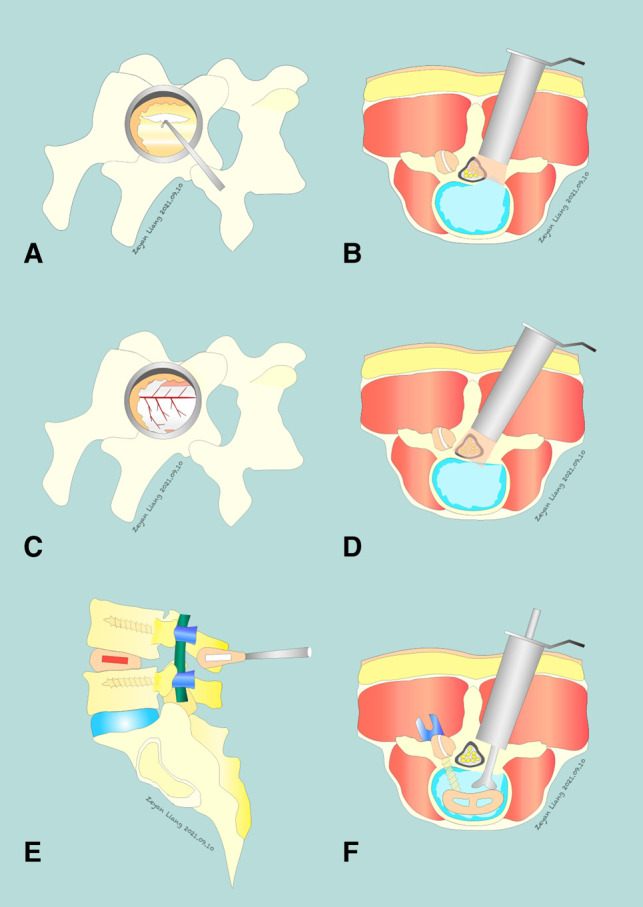
Comparison of PMTD and MIS TLIF Techniques. PMTD: (**A**) insertion of a nerve hook to start dissection of the ligamentum flavum (LF). (**B**) completing the ipsilateral decompression. (**C,D**) a complete removal of the LF is achieved and the dura is safely exposed. The contralateral exiting and traversing nerve roots may also be exposed if necessary. MIS TLIF: (**E,F**) an L4–5 MIS TLIF, a surgical option that includes a fusion procedure in addition to decompression.

After the target segment was located based on intraoperative fluoroscopy, a paraspinal incision (1.5–1.8 cm) was made, and the subcutaneous tissue and fascia were cut separately. The trocar and sequential tubular retractors will be placed paraspinally, under fluoroscopic control. The soft tissue on the surface of the lamina was bluntly separated step by step, and the lower margin of the lamina and spinous processes on the affected side of the upper vertebral body of the target segment was removed using a microdrill. After the ligamentum flavum was resected, the dura was fully exposed, and ipsilateral and contralateral decompression was performed (Figures 1A–1D). If necessary, the protruding or prolapsed nucleus pulposus tissue and some intervertebral nucleus pulposus were removed.

### Intervention: MIS TLIF

With fluoroscopic assistance, blunt separation was performed to expose the lamina and facet joints through the Wiltse space (33). The paraspinal tubular retractors were inserted, with the assistance of a microscope, the intervertebral disc tissue was fully processed, the osteophytes and hyperplasia soft tissue lesions of nerve compression were completely removed, the nerve root canal and lateral fossa were further expanded, and the compressors causing nerve root compression were completely removed. A similar procedure was performed on the other side if the same compression existed. An autologous bone fragment and an appropriate cage fusion device were implanted into the intervertebral space (Figures 1E,F).

### Outcome Measurement and Data Collection

Baseline information including sex, age, body mass index (BMI), comorbidities, ASA grade (34), target segment, clinical performance, duration of symptoms, relative slip distance of the vertebral body, preoperative Oswestry disability index (ODI) (35), and preoperative visual analog scale (VAS) (36) of the back and leg were collected to compare the baseline consistency between the two groups. The baseline and postoperative ODI, and baseline and postoperative VAS scores at 12 and 24 months were collected to compare the clinical efficacy. The VAS difference (i.e., ΔVAS) means the pre-operative VAS scores minus the final VAS scores. And The ODI difference (i.e., ΔODI) means the pre-operative ODI scores minus the final ODI scores. Surgical time, blood loss, length of incision, duration of hospital stay, hospitalization costs, incision infection, healing of operative incision, reoperation, and postoperative lumbar instability were used to compare clinical safety. Lumbar stability was defined postoperative hyperextension and flexion radiographs showed an angle difference of less than 10° between the upper and lower endplates of the affected segments or a transitional distance of less than 3 mm between the vertebral bodies.

### Statistical Analysis

Continuous variables are represented as mean ± standard deviation, and binomial distribution variables are expressed by frequency. An independent sample t-test was used to compare two sets of data that followed a normal distribution; otherwise, the Wilcoxon rank-sum test was used. Counting data were examined and analyzed using Chi-square nonparametric analysis. A *p*-value <0.05 indicated that the difference was statistically significant. For multivariate analyses, multivariate linear regression models were fitted for changes in ODI scores at 24 months (i.e., 24-month value - baseline value). All data were analyzed using SPSS 22.0.

### Sample Size

For the primary outcome, choosing a 5% noninferiority margin, a type 1 error of 0.05, and power of 0.80 gave a total sample size of 94 (20).

### Patient and Public Involvement

It was not appropriate or possible to involve patients or the public in the design, or conduct, or reporting, or dissemination plans of our research.

## Results

**Figure 2:**
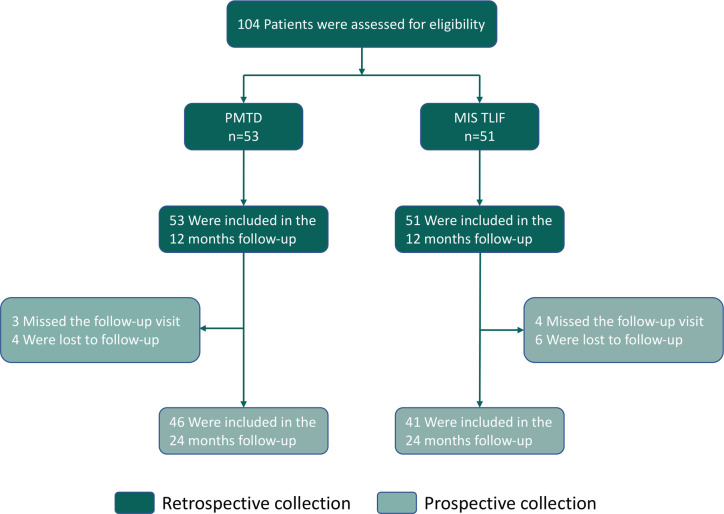
Study Flowchart.

A total of 104 patients with DLS-I-LSS were included in this study after screening based on inclusion and exclusion criteria. Fifty-three patients underwent PMTD, while the others underwent MIS TLIF. Figure 2 shows a flow chart of this study. The clinical data of patients who underwent PMTD or MIS TLIF for DLS-I-LSS were retrospectively collected at 12 months and prospectively collected at 12 to 24 months. The characteristics of the patients in the PMTD and MIS TLIF groups are shown in Table 2. There was a comparable equilibrium between the two groups (*p *> 0.05).

**Table 2:** Baseline characteristics of the patients.

Characteristics	PMTD	MIS TLIF	*p* value
Mean age (SD), years	62.06 (13.6)	59.94 (8.3)	0.34
Gender, No. (%)			0.052
Female	26 (50)	34 (67)	NA
Male	27 (50)	17 (33)	NA
Mean BMI (SD), kg/m^2^	23.70 (3.5)	24.30 (2.9)	0.34
Smoker, No. (%)	10 (19)	10 (20)	0.56
Comorbidities, No. (%)			
Diabetes mellitus	6 (11)	2 (4)	0.15
Hypertension	15 (28)	14 (28)	0.55
Coronary artery disease	1 (2)	2 (4)	0.49
ASA class III, No. (%)	19 (36)	17 (33)	0.48
Intermittent claudication, No. (%)	17 (32)	22 (43)	0.17
Symptom duration, No. (%)			0.35
<6 mos	4 (8)	6 (12)	NA
>6 mos	49 (92)	45 (88)	NA
Mean degree of vertebral slip (SD), mm	5.94 (2)	6.14 (2)	0.67
Segment underwent surgery, No. (%)			0.34
L3/4	6 (11)	7 (14)	NA
L4/5	40 (76)	32 (63)	NA
L5/S1	7 (13)	12 (23)	NA

*NA, no applicable*

### Oswestry Disability Index

**Figure 3:**
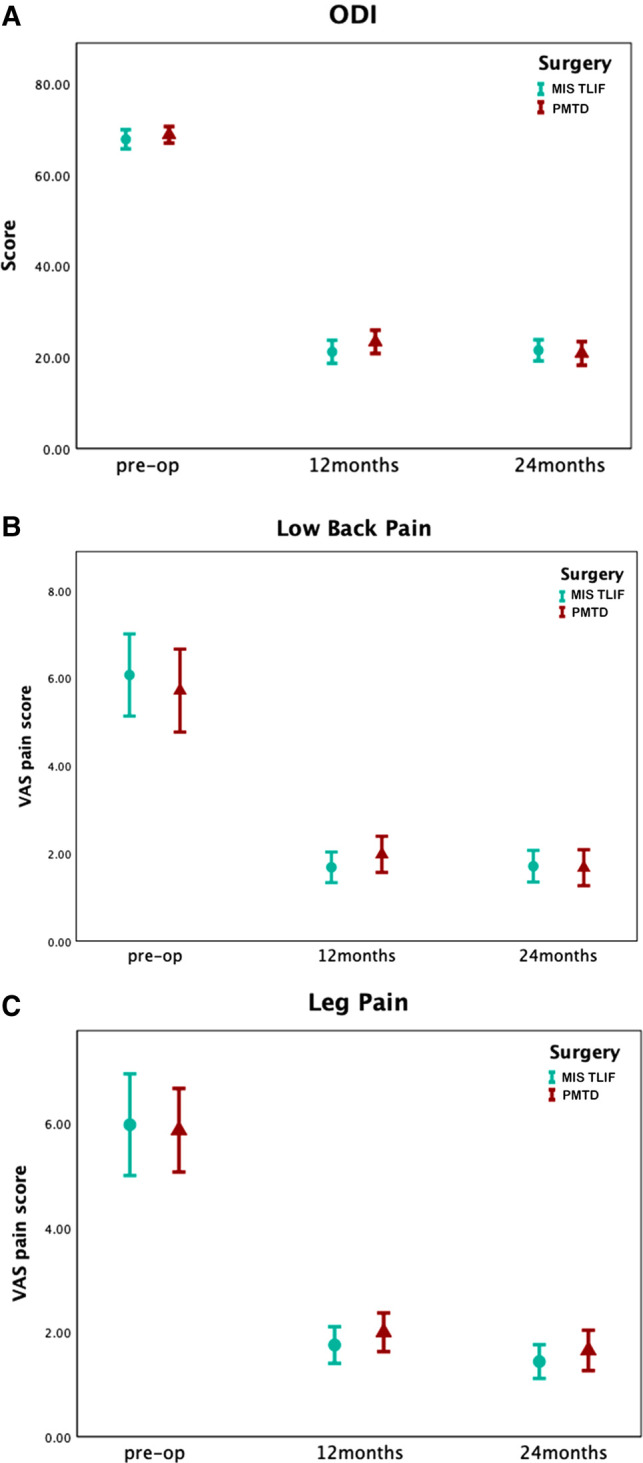
Preoperation, 12-month, and 24-month ODI and VAS following surgery for DLS-I-LSS. (**A**) Average ODI at preoperation, 12 months, and 24 months following surgery, by cohort. (**B**) Average VAS low back pain scores at preoperation, 12 months, and 24 months following surgery, by cohort. (**C**) Average VAS leg pain scores at preoperation, 12 months, and 24 months following surgery, by cohort. For both cohorts there were statistically significant improvements at 12 and 24-month follow-up, relative to preoperation, for ODI, VAS low back pain and VAS leg pain (*p* < 0.001, all comparisons).

In terms of ODI score, compared with PMTD (mean [SD] %, 45.73 [9.08]%), the ΔODI score of the MIS TLIF group at 12 months was 47.68 [10.13]%. The ΔODI score was 47.93 [10.52]% in the PMTD and 46.26 [10.05]% in the MIS TLIF group at 24 months. No significant differences were observed between the two groups at 12 months (PMTD minus MIS TLIF, 2.0%, 95% CI, −5.7% to 1.8%; *p* = 0.30) and 24 months (PMTD minus MIS TLIF, 1.7%, 95% CI, −2.7% to 6.1%; *p *= 0.45, Table 3). The postoperative ODI scores of both PMTD and MIS TLIF were significantly better than those before surgery (*p = *0*.*001, Figure 3A).

**Table 3:** Changes in ODI score and VAS score from baseline.

Variables	PMTD Group		MIS TLIF Group		Difference (95% CI)	*p* value
	No. of patients	Mean (SD)	No. of patients	Mean (SD)		
ΔODI score						
Preop	53	68.8 (6.0)	51	67.6 (6.4)	1.2 (−1.2, 3.6)	0.32
12 months	53	45.7 (9.1)	51	47.7 (10.1)	−2.0 (−5.7, 1.8)	0.30
24 months	46	47.9 (10.5)	41	46.26 (10.1)	1.7 (−2.7, 6.1)	0.45
ΔVAS low back pain score						
Preop	53	5.6 (3.2)	51	5.80 (3.2)	−0.2 (−1.4, 1.1)	0.79
12 months	53	3.8 (2.5)	51	4.10 (2.4)	−0.3 (−1.3, 0.6)	0.48
24 months	46	4.0 (2.6)	41	4.36 (2.3)	−0.3 (−1.4, 0.7)	0.55
ΔVAS leg pain score						
Preop	53	5.4 (3.0)	51	6.35 (2.9)	−0.9 (−2.1, 0.2)	0.11
12 months	53	3.6 (2.3)	51	4.37 (2.1)	−0.8 (−1.7, 0.05)	0.06
24 months	46	4.2 (2.4)	41	4.54 (2.5)	−0.3 (−1.4, 0.7)	0.55

### Visual Analog Scale

In terms of the ΔVAS lower back pain score at 12 months after surgery, there was no statistically significant difference between the two groups (PMTD minus MIS TLIF, −0.3 cm, 95% CI, −1.3 cm to 0.6 cm; *p *= 0.48; Table 3). Considering the ΔVAS lower back pain score at 24 months, the statistical analysis results showed no significant difference (PMTD minus MIS TLIF, −0.3 cm, 95% CI, −1.4 cm to 0.7 cm; *p *= 0.55; Table 3). The postoperative VAS lower back pain scores of both PMTD and MIS TLIF were significantly better than those before surgery (*p = *0*.*001, Figure 3B). Regarding the ΔVAS leg pain score at 12 months (PMTD minus MIS TLIF, −0.8 cm, 95% CI, −1.7 cm to 0.05 cm; *p *= 0.06; Table 3) and 24 months (PMTD minus MIS TLIF, −0.3 cm, 95% CI, −1.4 cm to 0.7 cm; *p *= 0.55), statistically significant differences were not observed. The postoperative VAS leg pain scores of both PMTD and MIS TLIF were significantly better than those before surgery (*p = *0*.*001, Figure 3C).

### Surgery Data and Adverse Events

All patients underwent surgery successfully without massive hemorrhage, dural tear, shock, or anesthesia accident during the operation. The operation time (PMTD minus MIS TLIF, −105.5 min, 95% CI, −129.6 min to −81.5 min; *p = *0*.*001; Table 4), estimated blood loss (−60.2 ml, 95% CI, −76.1 ml to −44.4 ml; *p = *0*.*001), length of incision (−4.6 cm, 95% −4.7 cm to −4.5 cm; *p *< 0.001), duration of hospital stay (−4.4 days, 95% CI, −6.0 days to −2.8 days; *p *< 0.001), and hospitalization costs (−33476.0 yuan, 95% CI, −36266.1 yuan to −30685.8 yuan; *p *< 0.001) in the MIS TLIF group were higher than those in the PMTD group, and the differences between the two groups were statistically significant. The adverse events were observed in the follow-up period, including incision infection, operative incision of healing, reoperation and lumbar instability. There were no statistically significant differences between two groups (*p *> 0.05, Table 4). The cumulative reoperation rates were 5.66% and 1.96% in the MIS TLIF and PMTD groups, respectively (*p *= 0.68).

**Table 4:** Surgery data and adverse events.

Variables	PMTD	MIS TLIF	Difference (95% CI)^a^	*p* value
**Surgery data**				
Mean operation time (SD), mins	191.9 (57.7)	297.43 (65.7)	−105.5 (−129.6, −81.5)	<0.001
Mean estimated blood loss (SD), ml	33.3 (26.9)	93.53 (51.2)	−60.2 (−76.1, −44.4)	<0.001
Mean length of incisions (SD), cm	2.0 (0.10)	6.63 (0.5)	−4.6 (−4.7, −4.5)	<0.001
Mean duration of hospital stay (SD), days	7.5 (3.3)	11.94 (4.9)	−4.4 (−6.0, −2.8)	<0.001
Mean hospitalization costs (SD), yuan^b^	22086.4 (5149.7)	55562.4 (8793.8)	−33476.0 (−36266.1, −30685.8)	<0.001
**Adverse events**				
Incision infection, No. (%)	0	2 (3.9)	NA	0.14
Operative Incision of Healing, No. (%)	5 (9.4)	7 (13.7)	NA	0.49
Reoperation, No. (%)	3 (5.6)	1 (1.9)	NA	0.68
Postoperative lumbar instability, No. (%)	3 (5.6)	0	NA	0.85

*Abbreviation: NA, not applicable.*

*^a^* *Calculated as PMTD minus MIS TLIF with 95% CI.*

*^b^The yuan is the basic unit of the renminbi, which is the official currency of the People’s Republic of China*.

### Multivariate Analysis

We incorporated surgery types, BMI, diabetes mellitus, degree of vertebral slip, and baseline ODI score into the multivariate analysis model to identify the prognostic factors affecting the efficacy of minimally invasive surgery. According to the multivariate model, BMI (*β* = −0.96, 95% CI, −1.4 to −0.48; *p *< 0.001), diabetes mellitus (*β* = −6.9, 95% CI, −12.7 to −1.0; *p *= 0.022), and baseline ODI score (*β* = 0.99, 95% CI, 0.75 to 1.2; *p *< 0.001) were the predictors of **Δ**ODI score for DLS-I-LSS at 24 months after MISS (Table 5). The model with these three variables correctly predicted the response in 50.5% of patients.

**Table 5:** Significant predictors of 24-month ΔODI score for DLS-I-LSS.^a^

Variables	*β*	95% CI	*p* value
BMI	−0.96	(−1.4, −0.48)	<.001
Diabetes mellitus	−6.9	(−12.7, −1.0)	.022
Baseline ODI score	0.99	(0.75, 1.2)	<.001

*^a^* *Adjusted R^2 ^= 0.505.*

## Discussion

It is now generally believed that lumbar spinal canal decompression and fusion treatment should be used when mobile DLS causes lumbar spine instability and lower back pain (37–39). Controversies remain regarding the surgical treatment of inactive DLS. Studies have shown that pure lumbar laminectomy may destroy the stability of the lumbar spine (40–42). However, with the development of minimally invasive spine surgery, PMTD technology has been used to treat spinal diseases such as LDH and LSS (18, 19). One of the problems that this study attempts to solve is the pros and cons of minimally invasive lumbar spinal canal decompression technology (i.e., PMTD) and minimally invasive lumbar fusion technology (i.e., MIS TLIF) in the treatment of inactive DLS-I-LSS. This cohort study included 104 patients with DLS-I-LSS to compare the efficacy and safety of PMTD and MIS TLIF. It involves the postoperative ODI score, VAS low back pain score, VAS leg pain score, surgical data, adverse events, and other key outcome indicators.

The VAS was used to assess the degree of lower back pain and leg pain before and after surgery to measure the degree of pain improvement. There was no significant difference in the ΔVAS score of leg pain and ΔVAS score of lower back pain between the PMTD and MIS-TLIF groups at 1 and 2 years after the operation. Therefore, the effects of PMTD technology and MIS-TLIF technology in improving patients with lower back and leg pain are similar. The results of Chan et al. also suggest that microdecompression and decompression plus fuison have similar effects in improving leg pain, while their results suggest that MIS-TLIF technology is better than PMTD technology in improving lower back pain (29). However, in a study by Chan et al. (29), the baseline characteristics of the population between the MIS decompression group and the MIS-TLIF group were inconsistent, which may be one of the reasons for the difference in results. In addition, Liang et al. (43) conducted a meta-analysis study, which included four randomized controlled trials and 13 observational studies, comparing the clinical efficacy of decompression fusion and simple decompression in the treatment of degenerative lumbar spondylolisthesis. The results showed that there was no significant correlation between fusion and improvement in the patients’ postoperative lower back pain VAS score and postoperative ODI score. The results of this study also showed that there was no statistically significant difference in ΔODI scores between the PMTD and MIS-TLIF groups at 1 year and 2 years after the operation. Therefore, compared with PMTD, MIS TLIF cannot improve the clinical benefit of patients’ symptoms and functional status within 2 years after surgery. In addition, the results of multiple linear regression analysis suggested that BMI, diabetes, and baseline ODI score were the main factors affecting the ΔODI score at 2 years after surgery. The lower ΔODI score in diabetic patients 2 years after surgery may be due to the overlap of the clinical manifestations of peripheral neuropathy and the symptoms of lumbar spondylosis, which reduces the recovery ability of nerve roots after surgery (44–46). Patients with a high BMI had a low degree of postoperative ODI improvement. The randomized controlled spine patient prognosis study trial (SPORT) showed that compared with non-obese patients, the improvement in postoperative ODI score of obese patients was significantly smaller (47). Patients with poor ODI scores at baseline will have the opportunity to achieve the greatest improvement after surgery, because patients with better functional status before surgery may be more susceptible to floor and ceiling effects (48).

Compared with the MIS-TLIF group, the PMTD group had a significantly shorter operation time, less intraoperative blood loss, smaller surgical incisions, shorter postoperative hospital stay, and lower total hospitalization costs. These results are consistent with conclusions of previous research (29, 49, 50). This is because the MIS-TLIF technology requires multiple paravertebral incisions to successfully insert the pedicle screw and bone graft fusion cage; therefore, the surgical incision is large, the amount of bleeding is large, and the fusion and internal fixation materials are involved, resulting in a significant increase in the cost incurred. In the PMTD group, there were three cases of lumbar spine instability occurring within 2 years after surgery and the patients returned to the hospital for internal fixation (one case had a lamina rupture due to a fall, and two cases were caused by a lamina fracture due to weight-bearing during the postoperative recovery period), and one case in the MIS TLIF group (adjacent segment degeneration). However, there was no significant difference in the cumulative reoperation rates between the two groups. Studies have reported that traditional decompression surgery alone has a significantly higher operation rate compared to the fusion group (14). Yavin et al. carried out a meta-analysis and found that there was a correlation between reoperative risk and fusion, which suggested careful patient selection is required (51). Compared with traditional decompression surgery, PMTD uses a paravertebral approach to bluntly separate the muscles, preserve the midline ligaments, and reduce muscle damage, which may reduce reoperation due to instability. Regarding the comparison of incision infection rate, fat liquefaction rate, and postoperative lumbar instability rate, the results were similar between PMTD and MIS TLIF. Based on the analysis of results of all the outcome indicators, the PMTD technique for the treatment of DLS-I-LSS can achieve curative effects similar to those of the MIS-TLIF technique, but it also has the advantages of low cost, short operation time, and a small incision. Therefore, PMTD technology has the potential to become a routine choice for the treatment of DLS-I-LSS.

## Study Limitations

This study analyzed in detail the clinical results of PMTD and MIS TLIF in the treatment of DLS-I-LSS, but there are still several limitations. (1) Although there was no significant difference in the baseline characteristics of patients between the PMTD group and the MIS-TLIF group, the study was a retrospective cohort study with a low level of evidence; (2) Although follow-ups were carried out for 1 and 2 years after the operation, the early follow-up data of the patients were missing, and early evaluation of the efficacy between the two groups could not be carried out; (3) Unlike MIS-TLIF, PMTD is a non-fusion technique. There are differences in the focus of the two techniques. Although the results of this study demonstrated that there was no significant difference between the groups two years after surgery, the comparison lacks rigor to some extent because longer follow-up results should be proposed in the future to demonstrate the applicability of the two techniques; (4) although both the PMTD and MIS-TLIF groups were quantitatively evaluated for pain and function, they did not evaluate outcomes such as satisfaction and quality of life. Therefore, to further verify the conclusions of the study, we conducted a multicenter prospective randomized controlled study (ChiCTR2100047365) to comprehensively assess patients’ early and long-term postoperative pain, functional status, quality of life, and other outcome indicators.

## Conclusion

Compared with MIS TLIF, PMTD in the treatment of patients with DLS-I-LSS showed no statistically significant differences in ODI improvement, VAS score for low back pain improvement, VAS score for leg pain improvement, and adverse event rates at 2 years after surgery; however, there was a shorter duration of hospital stay, shorter operation time, less blood loss, and lower hospitalization costs. BMI, presence or absence of diabetes, and baseline ODI score were the main influencing factors for the improvement of ODI in patients with DLS-I-LSS after minimally invasive surgery. The less extensive and less expensive treatment may be the primary surgical choice for most patients with DLS-I-LSS.

## Data Availability

The raw data supporting the conclusions of this article will be made available by the authors, without undue reservation.
